# Trends of HIV subtypes and phylogenetic dynamics among young men who have sex with men in China, 2009–2014

**DOI:** 10.1038/srep16708

**Published:** 2015-11-18

**Authors:** Zheng Li, Lingjie Liao, Yi Feng, Jing Zhang, Jing Yan, Cui He, Wei Xu, Yuhua Ruan, Hui Xing, Yiming Shao

**Affiliations:** 1 State Key Laboratory of Infectious Disease Prevention and Control, National Center for AIDS/STD Control and Prevention, Chinese Center for Disease Control and Prevention, Collaborative Innovation Center for Diagnosis and Treatment of Infectious Diseases, Beijing, China

## Abstract

To investigate the origins of HIV transmission and phylogenetic dynamics among men who have sex with men (MSM), a total of 1205 newly diagnosed HIV-infected 16–25 year-olds were recruited in 13 provinces across China between 2009 and 2014. Based on phylogenetic analyses of partial *pol* sequences, HIV-1 subtypes including CRF01_AE (45.3%), CRF07_BC (37.8%), subtype B (6.1%), and B’ (3.7%), as well as some other recombinants (7.1%) were identified. In addition to two distinct CRF01_AE clusters [cluster 4 (33.7%, 406/1205) and cluster 5 (7.1%, 85/1205)], we identified a new CRF07_BC cluster (cluster 1) (36.0%, 434/1205), which entered Chinese MSMs in 2004, and had been rapidly spreading since about 2004, which indicating the third wave of the HIV epidemic among the population. Moreover, two new clusters of CRF_01B recombinants were found in this study. The complexities of HIV subtypes and recombinants strongly supports the necessity for a comprehensive study about risk behaviors and their relationship with increasing HIV epidemic subtypes among the MSM group. Implementation and evaluation of comprehensive harm reduction strategies in Chinese MSM are urgently needed.

The first Chinese HIV epidemic was reported in 1989 in Yunnan^1^, a southwestern province located at the border between China and Burma along the major herion traffic, since then HIV transmission among Chinese drug users was rapidly increased^2^,^3^. By the end of 2007, the majority of HIV/AIDS cases resulted from heterosexual contact, beginning as a drug-driven epidemic and shifting to one driven predominantly by heterosexual contact in China^4^. More than 30 years have passed since the first HIV case was reported among men who have sex with men (MSM) in the USA^5^. MSM continues to be the major cause of new infection in the Americas, Western Europe, Oceania, and much of Asia^6^. Since 2003, China has launched an ambitious and comprehensive response to the national HIV/AIDS epidemic. This has reduced the high incidence of certain blood-borne infectious diseases^7^,^8^ and related risk behaviors among the high-risk IDUs and heterosexual population in China^9^,^10^,^11^,^12^. Due to lack of sufficient scientific data on HIV transmission, the potential high risk subgroup of MSM was neglected during this period in China. We conducted China’s first prospective cohort study among MSM in Beijing, which found that the incidence was 2.6, 3.7, and 8.1 per 100 PYs (person-years ) for HIV in 2006, 2007, and 2009, respectively^13^,^14^,^15^. Also, among the nationally reported HIV/AIDS cases, the proportion of MSM increased rapidly during 2005–2014, from 0.7% (total number of reported HIV/AIDS, 40,711) in 2005 to 10.0% (61,470) in 2009, and to 25.8% (103,501) in 2014^4^,^16^,^17^,^18^,^19^. Today, China has approximately 18 million MSM, making them one of the most important target risk populations for HIV prevention^20^. The objective of this study is to use phylogenetic and Bayesian molecular clock analyses to clarify the origin of transmission and divergence times of the epidemic strains among newly diagnosed HIV-infected MSM in China. We hope that this can provide deep insight into the evolutionary dynamics of HIV epidemics for future prevention.

## Results

### Identification of HIV-1 Subtypes among MSM in China

A total of 1205 HIV-1 nucleotide sequences of the 1.0-kb pol gene (HXB2: 2253–3278 nts) from newly diagnosed MSM between 2009 and 2014 from 13 Chinese provinces were determined and genotyped by phylogenetic tree analysis. As shown in Table 1, there were three known major Chinese circulating subtypes: CRF01_AE, 45.3%, CRF07_BC 37.8%, and subtype B_EU (U.S.-European origin) 6.1%, plus minor subtype B’ (Thailand) 3.7%, and other recombinants 7.1% in the MSM population. The total of CRF01_AE and CRF07_BC genotypes accounted for 83.1% (1001/1205) of the HIV-1 infections among MSM.

The chi-square trend test was used to compare the changes of HIV-1 subtypes over time. The proportion of subtype CRF01_AE decreased from 55.4% to 43.5% during 2009–2014 (P = 0.044), while at the same time, the proportion of subtype CRF07_BC increased from 25.6% to 40.1% (P = 0.016), and the proportion of subtype B_EU decreased from 16.0% to 3.8% (P < 0.0001). Other subtypes and recombinant strains can be seen in Table 1.

### Identification of seven independent clusters of HIV-1 strains among MSM in China

**Figure 1:**
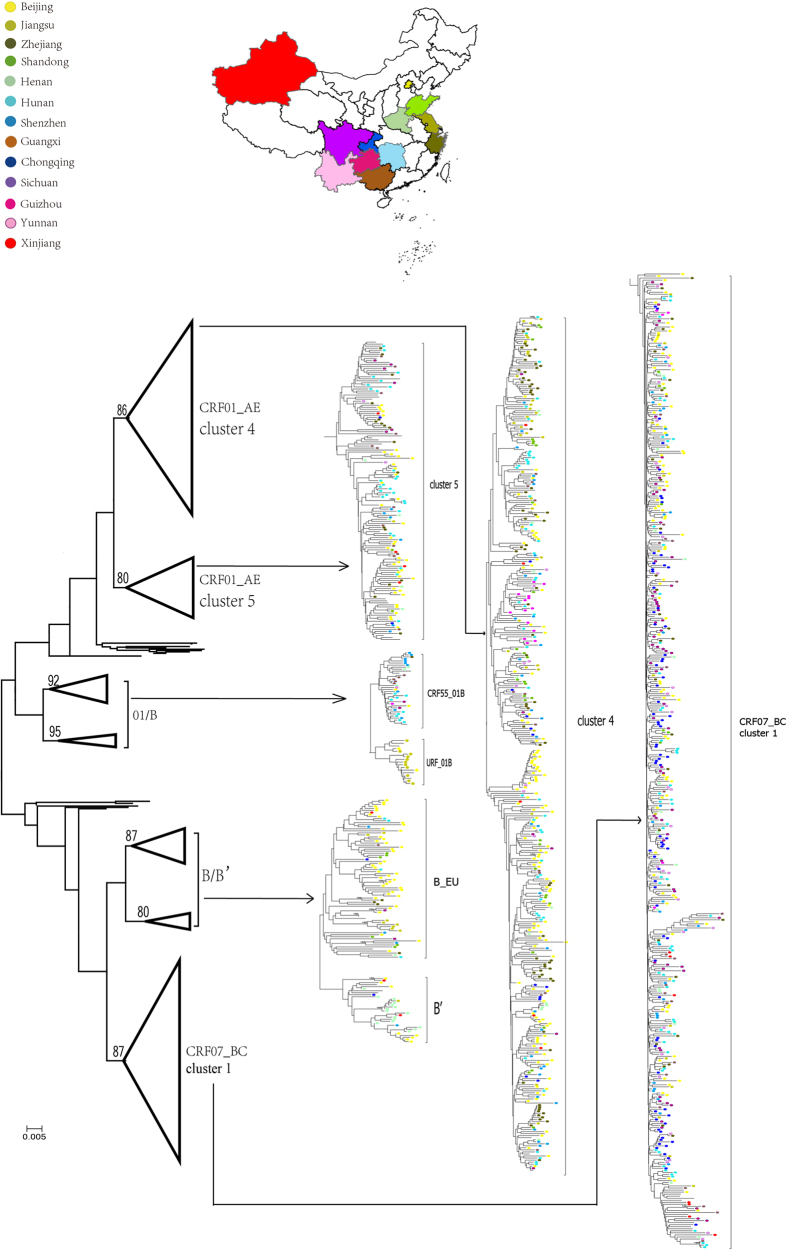
The phylogenetic tree constructed by PhyML 3.0 with the Maximum likelihood method, based on partial pol fragment. The geographic origin of each sequence is color-coded (see inset). The branch significance was analyzed by bootstrap with 500 replicates and inter-subject distances were calculated. Only bootstrap values above or equal to 70 are shown at the corresponding nodes. The map was generated by ArcGIS (http://www.esri.com/software/arcgis/arcgis-for-desktop/free-trial).

As shown in Fig. 1, the maximum-likelihood phylogenetic analysis identified seven distinct clusters among MSM, with high bootstrap confidence (≥80%). Subtype B was divided into cluster B_EU and cluster B’, and two distinct CRF01_AE clusters (clusters 4 and 5) which were previously reported by our group^21^. CRF07_BC formed a unique cluster (designated cluster 1) in the MSM population in China, which is distinct from the strains of other CRF07_BCs^22^. The proportion of CRF07_BC cluster 1 and CRF01_AE clusters 4 and 5 accounted for 36.0% (434 of 1205), 33.5% (404 of 1205), and 7.1% (85 of 1205) of HIV-1 infections among the MSM subjects, respectively. These 3 lineages of HIV-1 strains accounted for 76.6% (923 of 1205) of the MSM infections (Table 2), and were found in all provinces/cities in our study.

Beside the five major clusters, there are two clusters of CRF_01B among the MSM population, including a distinct cluster of CRF55_01B, the strains of which were mainly found in Shenzhen, Henan, and Hunan. We also found a new URF_01B cluster, which was only detected in Beijing and Jiangsu Province in our study, and the strains were clustered with the strains from Anhui Province.

**Figure 2:**
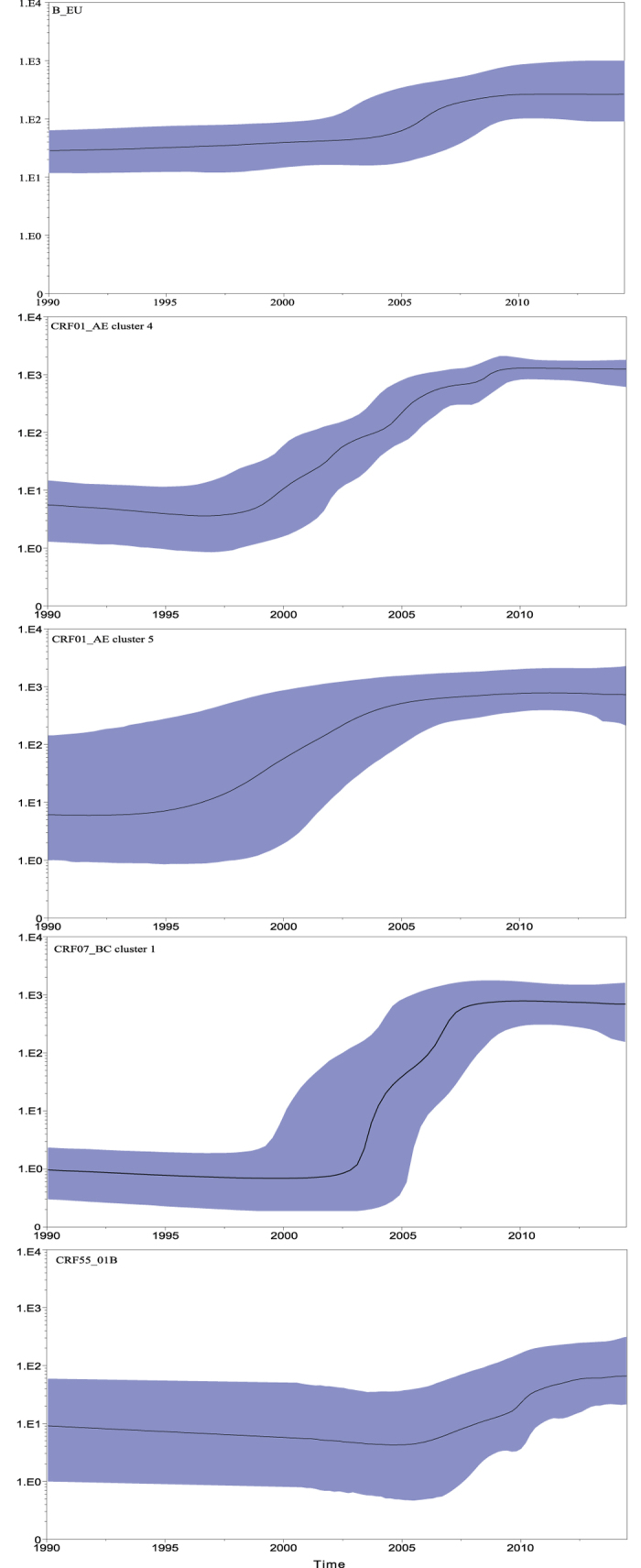
Baysian skyline plot was estimated to reconstruct the demographic history of the five major clusters (n ≥ 45) among MSM in China. The x axis is the time in units of years, and the y axis is equal to the effective population size. The thick solid line is the mean estimate and the 95% HPD credible region is shown by blue areas.

We reconstructed the epidemic history of the 5 major clusters (n ≥ 45) through Bayesian analysis, using the HKY model and the Log normal relaxed clock model. As shown in Fig. 2, the epidemic history of the five clusters was quite different. The strains of subtype B_EU first entered MSM in 1988, and were followed by CRF01_AE cluster 4, CRF01_AE cluster 5, CRF07_BC cluster 1, and CRF55_01B (Table3). The time of origin of CRF01_AE cluster 4 and cluster 5 was about the mid-1990 s. The Skyline plot result revealed that CRF01_AE cluster 4 had undergone significant growth during 1997–2008 and CRF01_AE cluster 5 had a rapid growth during 1996–2005. CRF07_BC Cluster 1 appeared late and then expanded fast during 2004–2008, replacing CRF01_AE cluster 4 and cluster 5, and became the biggest cluster.

## Discussion

In recent years, MSM have become the most significant increasing HIV-1 transmission route in China^16^,^17^,^18^,^19^. Although traditional epidemiological surveys focusing on MSM populations have been conducted, this is the first study that used bioinformatic techniques to track changes of HIV subtypes and phylogenetic dynamics among Chinese MSM. Our study found rapid changes in the proportion of HIV subtypes and seven independent clusters of HIV-1 strains in MSM. The multiple lineages of HIV viruses and newly recombinant strains that circulate in MSM indicates that the HIV epidemic among MSM is very complex^21^,^22^,^23^,^24^. Changes of HIV subtypes and phylogenetic dynamics and associated risk factors should be continuously tracked in order to provide scientific data for designing suitable prevention strategies and methods for facing the challenges of the fast spreading HIV epidemic among Chinese MSM.

The major finding in our study was that the new wave of the HIV epidemic among MSM was driven by the subtype CRF07_BC virus. CRF07_BC was first transmitted to MSM in about 2004, and has been the biggest cluster for over ten years. CRF07_BC originated in 1993 in China among IDUs in the western and southern provinces of China, including Xinjiang, Sichuan and Yunnan^23^. The CRF07_BC cluster was not discovered in the second nationwide molecular epidemiological investigation in 2002, and was only found in a limited number of male homosexuals in the third nationwide molecular epidemiological investigation in 2007^24^. Ten years ago, illicit drug use was uncommon among Chinese MSM^13^,^14^,^15^. Our previous study results showed that the use of nitrite inhalants was alarmingly prevalent among MSM in Beijing, and 47.3% of the participants used nitrite inhalants which were associated with high-risks of HIV infection in 2012^25^, while the proportion was only 0.8% during 2006–2007^13^. Drug abuse is common among MSM in Western countries and significantly contributes to HIV spread in that population^26^,^27^. Drug use can relax safer sex norms and increase unprotected anal sex and risk of acquiring HIV^24^. The biggest worry is that the history of severe HIV epidemics in developed countries could be repeated in China MSM, which will be due to non-injection drug use among MSM in China. Also, another study conducted by our laboratory indicated that the CRF07_BC recombinant strains, with relatively lower net charges in the V3 loop, exclusively utilize the CCR5 co-receptor for infection, exhibit slow replication kinetics in the primary target cells, and may be superior to other HIV-1 subtypes in initiating blood-borne infection in high-risk populations in China^28^. Given that few Chinese MSM inject drugs, future study needs to explore reasons driving the rapid transmission of the subtype CRF07_BC virus among MSM, which is the main subtype in Chinese IDUs.

Our study estimated that the subtype B_EU group viruses were first introduced into MSM in China in 1988, and then were replaced by other subtypes, such as CRF01_AE cluster 4 and cluster 5. The subtype B_EU virus might have originated in the United States and Europe, and entered China through travelers. This initial founder virus did not turn out to be the main HIV genotype in Chinese MSM.

Our study found that CRF01_AE cluster 4 and cluster 5 first entered MSM in about 1994 and 1995, respectively. Then, the subtype CRF01_AE virus rapidly and widely spread over time became the main HIV genotype in China MSM. CRF01_AE cluster 4 and cluster 5 were not discovered until the second nationwide molecular epidemiological investigation in 2002, and they were found only in a limited number of male homosexuals in the third nationwide molecular epidemiological investigation in 2007^21^,^22^,^23^,^24^. The national molecular epidemiologic survey provided evidence that all CRF01_AE clusters were introduced from Southeast Asia in the 1990 s, especially from Thailand, and the early transmission was limited to the eastern coastal areas and southwest border provinces, predominantly in heterosexual populations^21^,^24^. Under social and cultural pressure, most Chinese MSM hide their sexual orientation and many of them are married^13^,^14^,^15^. A high proportion of Chinese MSM have sex with women and MSM may have a bridging role in the spread of HIV between female sexual partners and their male sexual partners.

In addition, we observed two newly identified clusters of CRF_01B recombinant strains. One cluster was CRF55_01B, which was first identified from MSM in China. The CRF_01B recombinant strain first entered Chinese MSM in about 2004. It includes the subtype B fragment which is related to subtype B_EU, not the Thai B’ variant. Another recombinant strain, the CRF_01AE fragment is related to Thai strains of CRF01_AE, but not to those found in other MSM in China. Our study found that CRF55_01B circulated in most cities. The other cluster of CRF_01B (URF_01B) is a region-specific cluster identified in Beijing and Jiangsu, and shows distinct mosaic models with the isolated CRF_01B strains from foreign countries. Our previous study also found a high proportion of HIV subtypes and new recombinant HIV-1 in predominantly heterosexually infected populations in a sexually driven epidemic area of Yunnan Province, China^29^. Preventive intervention should be focused on multiple risk exposure behavior for reducing the HIV epidemic of those strains in the high risk group^29^,^30^,^31^,^32^.

This study has some limitations. The study sample sizes differed between cities. Some cities such as Beijing and Zhejiang yielded large samples, whereas other sites such as Guangxi and Yunnan yielded relative few subjects. Therefore, our estimate of HIV genotype distributions may be biased. Non-participants may have different characteristics, such as demographics and risk behaviors, which could lead to a selection bias. However, our serial cross-sectional studies systematically revealed the emergence of the CRF07_BC cluster, multiple lineages of HIV viruses, and newly recombinant strains circulating in Chinese MSM. Future study needs to clarify the possible risk factors that related with changes of HIV subtypes and phylogenetic dynamics in Chinese MSM. Despite the rapid changes of subtype incidence that occurred from 2009 to 2011, there were not many changes in the distribution of any of the subtypes during 2011–2014. In order to control the fast transmission of HIV among MSM, comprehensive prevention intervention programs have been conducted since 2010, including mass education, community outreach, condom promotion, rapid scale-up of HIV testing, and ART. The impact of such programs on HIV infection and related risk behaviors among MSM urgently needs to be evaluated, which can guide scientific evidence for implementing an effective means of reducing HIV transmission.

## Methods

### Ethics Statement

This study was approved by the China CDC Institutional Ethics Committee, and written informed consent was obtained from study participants. All experiments were performed in accordance with the approved guidelines and regulations and the experimental protocols were approved by the institutional review boards of China CDC.

### Study design and study subjects

A serial cross-sectional study was conducted from 2009 and 2014 in 13 provinces or cities, China. The subjects were enrolled from newly diagnosed HIV cases at the local Center for Disease Control and Prevention. Subjects eligible for study were between 16 and 25 years of age, newly diagnosed HIV-1 infected MSMs, and able to provide written informed consent. All study participants completed a questionnaire administered by trained interviewers in a private room. The research staff collected 8 mL of peripheral blood samples that were anti-coagulated with EDTA-3K. Plasma was separated within 6 hours after collection, tested for antibodies and HIV-1 RNA, and frozen at −80 °C for further analysis. This study was approved by institutional review board at the National Center for AIDS/STD Control and Prevention, Chinese Center for Disease Control and Prevention.

### Sequence Assembly

The QIAamp mini-viral RNA kit (Qiagen, Germany) was used to extract RNA from all plasma samples, according to the manufacturer’s instructions. The HIV-1 pol gene (protease 1–99 amino acids and part of reverse transcriptase 1–250 amino acids) was amplified, purified, and bi-directly sequenced in an ABI330XL sequencer (Applied Biosystems, Foster City, CA), according to previously published methods^33^.

The sequences were assembled with Sequencher 4.10.1 (Genecodes, Ann Arbor, MI) and then aligned with previously submitted sequences from our laboratory and other reference sequences from the Los Alamos database (http://www.hiv.lanl.gov/content/index) using the CLUSTAL X program (available at: http://www.clustal.org/clustal2/)^34^. The sequences were then manually edited using Bioedit 7.09 (available at: www.mbio.ncsu.edu/bioedit/bioedit.html). All positions that contained alignment gaps were removed. To exclude experimental contamination, similarities between the pol sequences in this study and the sequence database were analyzed by applying the Los Alamos HIV Database Web tools (http://www.hiv.lanl.gov).

### Phylogenetic analyses

PhyML 3.0 was used to estimate a maximum likelihood phylogenetic tree for sequences using the GTRtItG4 nucleotide substitution model^35^. Tree topologies were heuristically searched using the subtree pruning and regrafting procedure. The confidence of each node in the phylogenetic trees was determined using the bootstrap method with 1000 replicates. The final maximum likelihood tree was visualized using the program FigTree v1.3.1 (http://beast.bio.ed.ac.uk). The Recombinant Identification Program 3.0 (www.hiv.lanl.gov/content/sequence/RIP/RIP.html) of Los Alamos HIV database was used to verify recombinant sequences.

To estimate the evolutionary rate and the time of the most recent common ancestor (tMRCA) for the CRF01_AE and CRF07_BC lineages, we used BEAST v.1.8.0 under an uncorrelated log-normal relaxed clock model, GTRtG4 substitution model, and Bayesian skyline plot demographic model^36^,^37^,^38^. BEAST analysis was performed using Markov Chain Monte Carlo (MCMC) runs of 20 million generations and sampled every 1000 steps. The Bayesian MCMC output was analyzed using Tracer v1.5 (http://beast.bio.ed.ac.uk/Tracer).

### Statistical Analyses

All data were analyzed using SAS 9.2 software packages. The proportion of HIV subtypes over time was assessed using the chi-square trend test. P values < 0.05 were considered statistically significant.

### Sequence Data

The sequences have been deposited in GenBank with accession numbers KR822836 - KR824040.

## Additional Information

**How to cite this article**: Li, Z. *et al.* Trends of HIV subtypes and phylogenetic dynamics among young men who have sex with men in China, 2009-2014. *Sci. Rep.* **5**, 16708; doi: 10.1038/srep16708 (2015).

## Figures and Tables

**Table 1:** HIV-1 genotype distributions of different cross-sections (2009-2014).

Years	No. of cases	HIV-1 genotypes				
		B _EU	B’(TH)	CRF01_AE	CRF07_BC	Others^*^
Total (%)	1205	74 (6.1)	44 (3.7)	546 (45.3)	455 (37.8)	86 (7.1)
2009	125	20 (16.0)	2 (1.6)	68 (54.4)	32 (25.6)	3 (2.4)
2010	188	17 (9.0)	0 (0.0)	94 (50.0)	61 (32.5)	16 (8.5)
2011	203	9 (4.4)	5 (2.5)	86 (42.4)	89 (43.8)	14 (6.9)
2012	198	10 (5.1)	9 (4.5)	83 (41.9)	79 (39.9)	17 (8.6)
2013	199	7 (3.5)	16 (8.1)	88 (44.2)	77 (38.7)	11 (5.5)
2014	292	11 (3.8)	12 (4.1)	127 (43.5)	117 (40.1)	25 (8.5)
P		<0.0001	0.0013	0.044	0.016	0.23

^*^Others:

• URF01/B(2), URF01AE/BC(1)(2009). • C(3), URF01/B(11), URFAG(1), URFADG(1)(2010). • C(1), CRF08_BC(3), URF01AE/BC, URF01/B(9) (1)(2011). • C(1), URF01/B(10), URF01AE/BC(5), URF01/C(1)(2012). • URF01/B(8), URF01AE/BC(3)(2013). • C(1), URF01/B(19), URF01AE/BC(3), URF01/C/H(1), URF01/A(1) (2014).

**Table 2:** The distribution of HIV-1 genotypes among MSM in 13 provinces/cities across China.

Province/City	Total	B/B’			CRF01_AE				CRF07_BC			CRF_01B			Others
		n	B _EU	B’(TH)	N	cluster 4	cluster 5	other	n	cluster 1	other	n	CRF55_01B	URF_01B	
Total (%)	1205	118	75 (6.2)	43 (3.6)	546	404 (33.5)	85 (7.1)	57 (4.7)	455	434 (36.0)	21 (1.7)	59	45 (3.7)	14 (1.2)	27 (2.2)
Beijing	319	55	41 (12.9)	14 (4.4)	157	113 (35.4)	34 (10.7)	10 (3.1)	89	87 (27.3)	2 (0.6)	6	3 (0.9)	3 (0.9)	12 (3.8)
Shandong	46	7	6 (13.0)	1 (2.2)	31	29 (63.0)	2 (4.3)	0 (0.0)	7	7 (15.2)	0 (0.0)	0	0 (0.0)	0 (0.0)	1 (2.2)
Jiangsu	72	9	8 (11.1)	1 (1.4)	37	35 (48.6)	1 (1.4)	1 (1.4)	15	14 (19.4)	1 (1.4)	11	0 (0.0)	11 (15.3)	0 (0.0)
Zhejiang	133	6	6 (4.5)	0 (0.0)	91	74 (55.6)	7 (5.3)	10 (7.5)	28	28 (21.1)	0 (0.0)	3	3 (2.3)	0 (0.0)	5 (3.8)
Shenzhen	87	4	3 (3.4)	1 (1.1)	41	33 (37.9)	6 (6.9)	2 (2.3)	28	28 (32.2)	0 (0.0)	12	12 (13.8)	0 (0.0)	2 (2.3)
Henan	55	17	1 (1.8)	16 (29.1)	17	12 (21.8)	5 (9.1)	0 (0.0)	17	16 (29.1)	1 (1.8)	4	4 (7.3)	0 (0.0)	0 (0.0)
Hunan	166	11	6 (3.6)	5 (3.0)	81	50 (30.1)	19 (11.4)	12 (7.2)	61	57 (34.3)	4 (2.4)	13	13 (7.8)	0 (0.0)	0 (0.0)
Xinjiang	24	3	1 (4.2)	2 (8.3)	11	6 (25.0)	3 (12.5)	2 (8.3)	7	5 (20.8)	2 (8.3)	1	1 (4.2)	0 (0.0)	2 (8.3)
Chongqing	96	2	1 (1.0)	1 (1.0)	15	11 (11.5)	1 (1.0)	3 (3.1)	79	78 (81.3)	1 (1.0)	0	0 (0.0)	0 (0.0)	0 (0.0)
Sichuan	41	0	0 (0.0)	0 (0.0)	11	7 (17.1)	1 (2.4)	3 (7.3)	28	26 (63.4)	2 (4.9)	1	1 (2.4)	0 (0.0)	1 (2.4)
Guangxi	60	2	1 (1.7)	1 (1.7)	27	14 (23.3)	6 (10.0)	7 (11.7)	25	19 (31.7)	6 (10.0)	5	5(8.3)	0 (0.0)	1 (1.7)
Guizhou	84	2	1 (1.2)	1 (1.2)	12	6 (7.1)	0 (0.0)	6 (7.1)	66	64 (76.2)	2 (2.4)	2	2 (2.4)	0 (0.0)	2 (2.4)
Yunnan	22	0	0 (0.0)	0 (0.0)	15	14 (63.6)	0 (0.0)	1 (4.5)	5	5 (22.7)	0 (0.0)	1	1 (4.5)	0(0.0)	1 (4.5)

**Table 3:** Estimated Substitution Rates and Dates for Transmission Clusters.

Cluster	No. of sequences	Rate of evolution (year-1)	Date of origin (tMRCA)
B_EU	75	1.37 × 10^−3^	1988 (1985–1992)
CRF01_AE cluster 4	406	3.16 × 10^−3^	1994 (1991–1999)
CRF01_AE cluster 5	85	1.58 × 10^−3^	1995 (1992–2000)
CRF07_BC cluster 1	434	1.51 × 10^−3^	2004 (2002–2005)
CRF55_01B	45	1.94 × 10^−3^	2004 (2000–2007)
